# Peptidome: Chaos or Inevitability

**DOI:** 10.3390/ijms222313128

**Published:** 2021-12-04

**Authors:** Irina Lyapina, Vadim Ivanov, Igor Fesenko

**Affiliations:** Department of Functional Genomics and Proteomics of Plants, Shemyakin and Ovchinnikov Institute of Bioorganic Chemistry Russian Academy of Sciences, 117997 Moscow, Russia; amadeynemez@gmail.com (I.L.); ivavt@ibch.ru (V.I.)

**Keywords:** peptidomes, biologically active peptides, peptidome generation, protein precursors

## Abstract

Thousands of naturally occurring peptides differing in their origin, abundance and possible functions have been identified in the tissue and biological fluids of vertebrates, insects, fungi, plants and bacteria. These peptide pools are referred to as intracellular or extracellular peptidomes, and besides a small proportion of well-characterized peptide hormones and defense peptides, are poorly characterized. However, a growing body of evidence suggests that unknown bioactive peptides are hidden in the peptidomes of different organisms. In this review, we present a comprehensive overview of the mechanisms of generation and properties of peptidomes across different organisms. Based on their origin, we propose three large peptide groups—functional protein “degradome”, small open reading frame (smORF)-encoded peptides (smORFome) and specific precursor-derived peptides. The composition of peptide pools identified by mass-spectrometry analysis in human cells, plants, yeast and bacteria is compared and discussed. The functions of different peptide groups, for example the role of the “degradome” in promoting defense signaling, are also considered.

## 1. Introduction

Peptides play key roles in numerous processes, including growth regulation, stress response, and immune signaling in all living organisms [1,2,3,4,5,6]. Systemic studies of the biodiversity of peptides, which began in the early 1990s, demonstrated modest progress in the first several years owing to the limitations of the available analytical techniques. For example, in 2005–2006, the list of peptides of studied samples usually contained not more than a few hundred discrete peptide sequences [7,8,9]. However, the rapid development of modern mass-spectrometry analysis coupled with the explosive growth of genetic data banks has led to the considerable expansion of the list of characterized native peptidomes. Tens of thousands of peptides that significantly differ in their origin, function and properties have been identified in the tissue and biological fluids of multiple organisms [10,11,12,13,14,15,16,17]. Even though the peptidomes of prokaryotic and eukaryotic cells comprise thousands of peptides, the majority of them are generated during protein degradation [18]. These peptides are referred to as the “protein degradome” [19] and perhaps are no more than cell “trash” remaining after unspecific proteolysis. The bulk of the intracellular “protein degradome” appears to be generated by the proteasomal degradation of functional proteins into 5–22 amino acid (aa) peptides, followed by oligopeptidases cleavage [20,21]. 

In addition to peptides from functional proteins, some peptide hormones, antimicrobial peptides, etc., released from specific protein precursors by proteolytic cleavage can be found in peptidomes. In addition, the translation of thousands of small open reading frames (smORFs; <100 codons) located on long non-coding RNAs (lncRNAs) or mRNAs was confirmed experimentally and, therefore, is another source of peptides in cellular and secreted peptidomes [22,23,24,25,26,27,28]. However, the abundance of these groups of peptides, their half-life and degradation mechanisms are still poorly understood [29,30,31,32].

Although intracellular peptides were first described in the 1950s [33,34], our understanding of their possible function is still insufficient. For example, peptides presented by major histocompatibility complexes (MHC) are generated from cellular proteins and play a role as antigens in self-recognition [35]. It was recently shown that previously unannotated “non-canonical” proteins generate major histocompatibility complex I (MHC I)-bound peptides 5-fold more effectively than annotated proteins [36]. As another example, the contribution of alternative open reading frames (altORFs) [37] to shaping the composition of intracellular or secreted peptidomes is still unknown. The translation of such altORFs may be higher than longer protein-coding ORFs [38] and their degradation can make a significant contribution to native peptidomes. In recent years, a growing body of evidence has emerged that biologically active peptides may be hidden in the sequences of functional proteins, and in most cases, the functional activity of these peptides may differ from the respective proteins. Such peptides are called “cryptides” or cryptic peptides [39]. In plants, only three cryptides involved in the immune response have been identified [40,41,42]. There are more examples of mammalian cryptides derived from proteins such as hemoglobin [43,44,45,46], mitochondrial proteins [47], proteasome [48] and others [49].

The antigen presentation of peptides derived from cellular proteins in mammals is an example of the complex roles of peptidomes in promoting cellular homeostasis and the response to external stimulus [50,51]. Furthermore, recent studies have shown that the innate immune system in animals is based on the perception of “proteinaceous” signals both from pathogens and from host cells [52,53,54,55,56]. Plants have a similar system of release and recognition of damage-associated molecular patterns (DAMPs), as well as pathogen-associated molecular patterns (PAMPs) [57]. The receptors involved in this type of danger signaling have been found in a broad range of organisms, from insects and mammals to plants [58]. Stress conditions influence the composition of peptide pools, thereby, resulting in the release of potential antimicrobial agents from functional proteins [15,59,60,61]. This rapid stress response at the peptidome level based on protein degradation can be considered as a concerted action of the whole peptidome.

Thus, the peptidomes of tissue and biological fluids have a complex nature and should not be considered as just a set of independent functional and non-functional peptides, but as a self-complementing biologically active matter. In this review, we summarize our knowledge about the generation of different types of peptides, their precursors and biological function. In addition, we analyzed selected peptidome datasets (Table 1) from plants [15,16,62], bacteria [63], humans [12,64] and yeast [65] to identify some common trends in their composition and physicochemical properties.

## 2. Mechanisms of Peptide Generation from Protein Precursors

**Figure 1:**
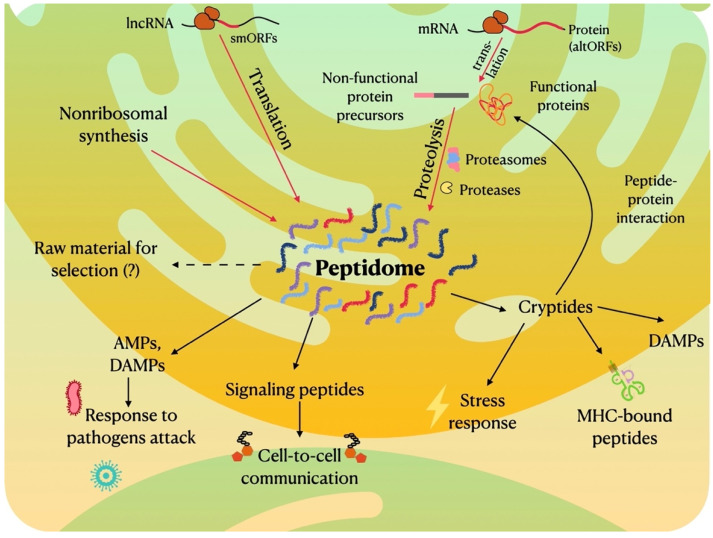
General scheme of generating and functioning pathways of naturally occurring peptidomes.

The known mechanisms of peptide generation include the specific proteolysis of functional or non-functional proteins by different classes of proteolytic enzymes (proteases), ubiquitin-dependent or independent digestion by proteasomes and the translation of small ORFs into peptides (Figure 1). Nonribosomal peptides [66] are outside the scope of this review.

### 2.1. The Protease-Specific Cleavage of Protein Precursors

The release of peptide hormones during the protease-specific cleavage of corresponding protein precursors at a specific site is well-studied in many organisms [67,68]. The architecture of these, mainly unfunctional, protein precursors is quite similar among plants, mammals, bacteria and yeast. They contain an N-signal sequence, a cleavage site for a certain protease and a functional sequence [69,70,71,72]. Interestingly, the precursors of some human peptide hormones contain other protein-coding sequences along with the bioactive peptides, as in the case of vasopressin and oxytocin [73,74]. Apparently, the specific proteolytic cleavage results in the generation of “peptide ladders” encompassing bioactive amino acid motifs [12,75]. 

Based on catalytic active sites, all known proteases are divided into five families, such as aspartyl-, cysteine-, metallo-, serine-, and threonine proteases that are well established among different organisms [18]. It has been shown that serine proteases (subtilases) play a pivotal role in the release of peptide hormones in plants and mammals [16,70,76]. For example, subtilase S1P (SITE 1 PROTEASE)/SBT6.1 is responsible for the biogenesis of the RGF/GLV/CLEL and RALF peptide hormones in plants [77]. In mammals, seven subtilisin/kexin-like endoproteases named prohormone convertases (PCs) are responsible for the release of neuropeptides [78]. Precursors of human growth factors are reported to be embedded in the membranes of vesicles and bioactive peptides can be released by extracellular proteases, such as serine proteases, upon the merging of vesicles with plasma membranes [79]. Vasopressin and oxytocin are derived from their precursors by subtilisin-like prohormone convertases SPC3 [73]. 

Recently, it was also shown that metalloproteases (referred to as a “sheddases”) take a considerable part in the process named “ectodomain shedding” in animals [80]. Through this process, many membrane-bound peptides such as growth factors and cytokines are released in specific conditions [81]. “Shedding” also contributes to signal transmission, liberating intracellular parts of transmembrane proteins into cytoplasm [82].

Another important protease family involved in the release of bioactive peptides is the cysteine proteases. In plants, this family includes papain-like proteases and metacaspases and participates in the release of some immune peptides [83,84,85,86]. In mammals, cysteine protease cathepsin V produces the neuropeptides enkephalin and neuropeptide Y (NPY) [87].

However, the role of proteases in shaping the whole intra- and extracellular peptidomes still remains poorly studied. Presumably, proteases make a significant contribution to the shaping of the secreted peptidome [16,68]. It was observed that treatment with stress phytohormones triggered the increase in activity of subtilisin-like serine protease, such as P69B, and papain-like cysteine proteases, such as PIP1 and some others in plants [83,88]. Analysis of the N- and C- ends of peptides in the plant secretome under stress conditions also showed the predominance of serine- and metalloproteases [16]. Serine, metalloprotease and cysteine protease activity have also been shown in secreted peptidomes of human bodily fluids [12,68]. 

### 2.2. The Proteasomal Degradation of Functional Proteins

The proteasomal degradation pathway apparently plays a major role in the formation of intracellular protein “degradome”. Proteasomes are multisubunit complexes that are responsible for the degradation of functional proteins in cells. Proteasomal subunits possess caspase-like (β1), trypsin-like (β2) and chymotrypsin-like (β5) proteolytic activities and degrade proteins into 3–25 aa peptides that are subject to further degradation by proteases [20,21]. In a recent study, several thousand peptides associated with proteasomes were identified in human cells [64]. These data were in line with previously published results showing that specific reversible and irreversible proteasome inhibitors, such as bortezomib and epoxomicin, influence the process of the generation and degradation of intracellular endogenous peptides in mammalian cells [89,90,91,92]. These studies clearly showed that thousands of intracellular peptides are a by-product of proteasomal degradation. However, no correlation was found between the number of identified peptides and the abundance of the corresponding precursors in different organisms [64,93]. Nevertheless, the abundance of the intracellular peptides can be influenced by different factors. For example, the stimulation of HEK293 cells with the cytokines TNF-α and IFN-γ for 24 h resulted in changes in the abundance of numerous proteasome-associated peptides [64]. Upon stress conditions, proteasomes in human cells tended to cleave protein precursors of known self-antigens such as histones [64].

The peptides released by proteasomes undergo further hydrolysis to amino acids [94,95,96]. The key players in this process are oligopeptidases, such as thimet oligopeptidase (THOP1, EC 3.4.24.15; EP24.15). For example, THOP1 metabolizes bradykinin [97,98], neurotensin [99], opioid peptides [100] and angiotensin [101]. The overexpression or knockdown of THOP1 in HEK293T cells resulted in a decrease in the abundance of some intracellular peptides [102,103]. In addition, peptidomic analysis of specific brain regions in THOP1 knockout mice revealed differences in the abundance of some intracellular peptides [104]. However, the mechanisms of intracellular peptide degradation are less studied in other organisms. For example, peptides originating from chloroplast proteins were sequentially degraded by prolyl (PreP) and organellar (OOP) oligopeptidases to 2–5 aa fragments and then to single amino acids by aminopeptidases M1, M17-10, M17-20 and M18 [105]. 

**Figure 2:**
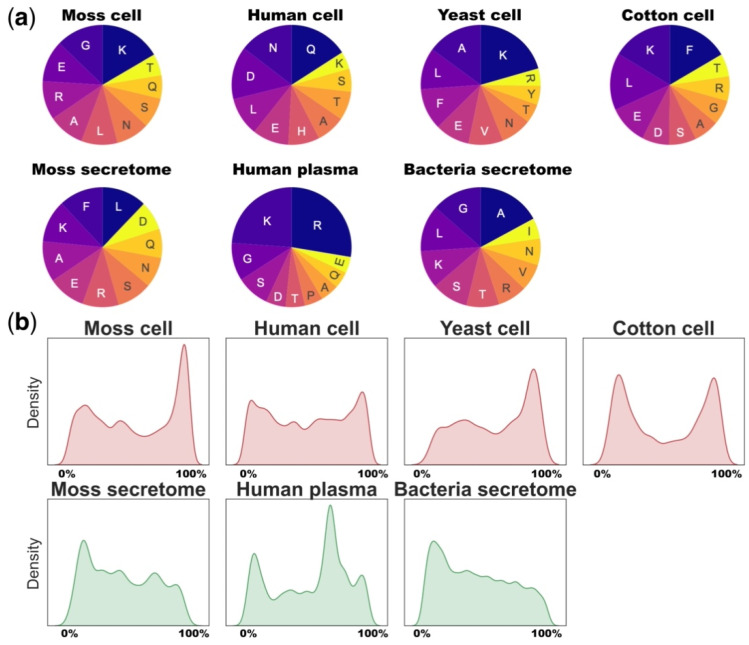
The peptidome degradation patterns. (**a**)—Pie chart showing the top 10 amino acids at the C-terminal positions of intracellular peptides from moss [15], human [64], yeast [65], cotton [62] and extracellular peptides from human plasma [12], moss [15,16] and bacteria [63]; (**b**)—Density plot showing the distribution of MS-identified peptides across the precursor lengths in peptidomic datasets from moss [15,16], human [12,64], yeast [65], cotton [62] and bacteria [63]. The positions of each identified peptide were normalized to protein lengths and represented as percentages. The steps of visualizing and analysing the data in all figures are available in the GitHub code repository: https://github.com/IgorFesenko/Peptidome_review.

It is well established that proteasomal subunits target specific amino acid motifs enriched in negatively charged residues (D, E; caspase-like), hydrophobic residues (W, F, M, Y; chymotrypsin) and positively charged residues (R, K; trypsin-like) [64]. This specific cleavage results in specific compositions of intracellular peptide pools. For example, C-ends of the proteasome-associated peptides were consistent with caspase-like and chymotrypsin-like activities of proteasomes, but not with the trypsin-like activity [64]. Our analysis of terminal amino acids of different peptidome datasets showed that lysine (K) and arginine (R) were among the most represented at C-terminal peptide cleavage sites of the considered peptidomes, except the specific proteasome-associated peptidome of human cells. Wolf-Levy et al. suggest that this is owing to either biological or technical reasons (Figure 2a) [64]. This might indicate that trypsin-like protease activity makes a significant contribution to the shape of native peptidomes. The overall discrepancy in C-terminal amino acids in different datasets may be owing to various reasons, such as nonspecific proteolysis, cutting up the ends of peptides by exopeptidases, technical features of the isolation method or biases of MS analysis technology (Figure 2a). 

Most of the known plant peptide hormones have been reported to originate from the C-terminus of their respective protein precursor [106,107,108]. On the other hand, peptide hormone precursors from humans and animals often have a multi-domain structure, generating multiple identical, homologous or entirely different functional peptides from different parts of a single precursor [50,70,71,109,110]. It has been previously shown that identified peptides are not evenly distributed across the protein lengths and native intracellular peptidomes are often the N- or C-terminal fragments of the corresponding protein precursors [10]. 

To determine whether peptides tend to originate from precursor ends, we calculated the frequency of their occurrence across the length of the corresponding proteins in different peptidomes. These data were presented as density plots showing the probability distributions of these frequencies. Indeed, the comparison of different datasets showed that peptides released from the C- or N-terminus tended to be more represented in the intracellular or extracellular peptidomes than we would expect in the case of random cleavage of proteins (Figure 2b). Overall, the degradation patterns of protein precursors from different datasets are similar with the predominance of C-terminal peptides in intracellular and N-terminal peptides in extracellular peptidomes. The unique patterns of human plasma and cotton peptidomes may reflect the technical variability during peptides isolation or the specificity of plant root tissue (Figure 2b).

## 3. Properties of Mass-Spectrometry-Based Peptidomes

**Figure 3:**
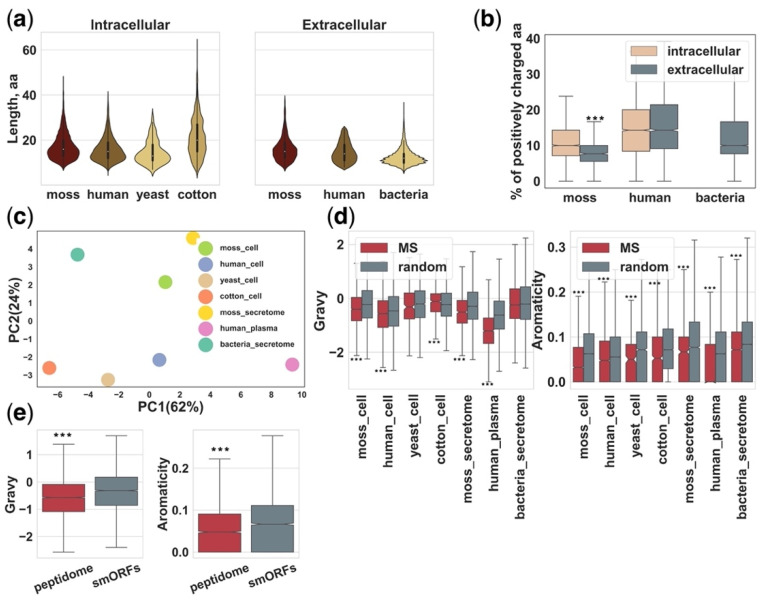
The properties of peptidomes identified by mass-spectrometry analysis. (**a**) The lengths distribution of intracellular peptides from moss [15], cotton [62], human [64], yeast [65] and extracellular peptides from human plasma [12], moss [15,16] and bacteria [63] datasets. (**b**) The percent of positively charged amino acids in peptides from different datasets. All calculations were performed by iFeature tool [119]. (**c**) Principal component analysis of the physicochemical properties of composition, transition and distribution (CTD) of the peptidomes from different organisms. The 2D plot demonstrates separation of peptidome’s amino acid composition in different datasets and clusterization of intracellular and extracellular datasets. All calculations were performed by iFeature tool [119]. (**d**) A comparison of the GRAVY indices and the proportion of aromatic acids between MS-identified peptides and sets of random peptides from the same proteins. The sets of random peptides were separately generated from the corresponding precursors for each dataset. All calculations were performed by Biopython [120]. (**e**) Comparison of the GRAVY indices and the proportion of aromatic acids in mass-spectrometry identified peptides from intracellular proteins [64] and small open reading frames [37] from human. *** *p* < 10–5 Mann–Whitney U-test. The steps of visualizing and analyzing the data in all figures are available in the GitHub code repository: https://github.com/IgorFesenko/Peptidome_review.

Our understanding of intra- and extracellular peptidomes is tightly coupled with mass-spectrometry (MS) analysis of extracted peptides from tissue and biological fluids. In selected datasets (Table 1), the median length of MS-identified endogenous peptides ranges from approximately 11 to 18 residues and is similar across cellular and secretome datasets from different organisms (Figure 3a). 

Peptidomic analysis usually includes the following steps: sample collection, peptide extraction, fractionation, LC-MS/MS analysis, peptide identification and data mining [111]. Therefore, cellular or extracellular peptide pools can be represented as a juxtaposition of peptides generated in tissue or biological fluids in native conditions and the result of postmortem and/or extraction artifacts [112]. In addition, methods of sample preparation [12] and LC-MS/MS analysis can contribute to the predominant identification of peptides with certain physicochemical properties. However, the physicochemical properties of MS-identified peptide pools are poorly studied. It has been previously shown that peptides from the secretome of the moss *Physcomitrium* (*Physcomitrella*) *patens* tended to have less positively charged amino acids than intracellular peptides and contain more hydrophobic amino acids (Figure 3b) [15]. This fact could reflect the properties of membrane or secreted proteins [113] that are, apparently, the main source of peptides in the secretome. However, further experiments and efforts are needed to shed light on this question. 

Indeed, the proteome structure, methods of peptide isolation and identification seem to influence the amino acid composition of MS-identified peptidomes (Figure 3c). These differences can impede the comparative analysis of peptidome datasets from different organisms. In a recent study, peptidomic analysis of HK-2 cells treated with TGF-β1 revealed that the GRAVY indices, indicating the hydrophobicity of the peptide sequence [114], of significantly altered endogenous peptides were mostly below zero, suggesting that most of them were hydrophilic peptides [92]. It seems that the identification of less hydrophobic peptides than expected by chance might be a general trend in peptidomic studies. For example, the GRAVY indices and the proportion of aromatic amino acids were significantly lower in almost all analyzed peptidomic datasets in comparison with sets of random peptides generated from the same proteins by chance (Figure 3d). This could reflect biological trends in the composition of cellular and secretome peptidomes or be a result of biases in sample preparation and LC-MS/MS analysis. For example, very hydrophilic short peptides can be lost during C18 separation [115]. 

It can be suggested that different groups of precursors can generate peptides with specific properties. For example, the hydrophobicity of human MS-identified peptides from smORFs was significantly higher than those of proteins (Figure 3e). This is in line with recent studies that show that novel adaptive smORFs are prone to containing transmembrane domains [116,117,118]. Therefore, our view of naturally occurring peptidomes, based on MS analysis, may be biased toward peptides with certain physicochemical properties.

## 4. The Functional Protein Precursors of Peptides

Are there specific sets of protein precursors that are the main source of naturally occurring peptides? Are there similar degradation patterns of these precursors in different organisms? According to a conservation analysis of yeast *Saccharomyces cerevisiae* and mammalian protein precursors, at least 30% of the yeast precursors had orthologs in mammalian peptidomes, such as ribosomal proteins, heat shock proteins and proteins involved in metabolic pathways [65]. The degradation patterns of some of these precursors, for example acyl-Co-A-binding protein, were similar [65]. A comparison of the cellular location of human and yeast precursors showed that most of the identified peptides originated from cytoplasmic proteins and mitochondrial proteins [65]. In addition, a substantial portion of precursors in yeast and human cells constitute nuclear proteins [65]. The GO enrichment analysis of precursors showed that most of them were involved in metabolism, the maintenance of reduction/oxidation balance, translation/protein synthesis, chaperone/protein folding, protein/vesicle trafficking and proteolysis [65]. 

In plant green tissue, a significant portion of peptides come from chloroplast and mitochondrial proteins, as was shown in the moss *Physcomitrium (Physcomitrella) patens* peptidomes [15,16,121,122]. In addition, intracellular peptides are derived from proteins involved in photosynthesis, the Calvin cycle, glycolysis and sucrose biosynthesis in *P.patens* [15]. Precursors of peptides extracted from the roots of *Gossypium arboreum* after inoculation with *Verticillium dahliae* also included pathogenesis-related protein STH2, eukaryotic aspartyl protease family protein and histone H2A [62]. Thus, a significant portion of intracellular peptides in different organisms is released from organellar proteins and some housekeeping proteins. 

Besides intracellular peptidomes, extracellular peptides have been analyzed in a number of studies [12,15,16,63]. The precursors of *Lactococcus lactis* bacterial secreted peptides belonged to extracellular, intracellular and transmembrane proteins [63]. Peptides were also released from a stable pool of precursor proteins, and the presence of peptides from intracellular proteins in the extracellular space were not related to the lysis process [63]. Among cytoplasmic protein precursors, proteins such as acetolactate synthase, bifunctional acetaldehyde CoA/alcohol dehydrogenase and ribosomal protein RpsT have been identified. Peptides were also released from cytoplasmic proteins, the secretion of which has been shown for many bacteria, such as glyceraldehyde-3-phosphate dehydrogenase, enolase, elongation factor TU, chaperone protein DnaK and pyruvate dehydrogenase E1 component beta subunit [63].

According to a recent study, the majority of precursors in the human plasma peptidome belong to secreted or cell membrane proteins [12]. In addition, the precursor proteins were from mitochondria, Golgi apparatus, endoplasmic reticulum and different vesicles. The GO enrichment analysis showed that these precursor proteins participate in muscle filament sliding, platelet degranulation/activation, exocytosis, glucose metabolic process and secretion by the cell [12]. Among identified peptides, known peptide hormones and growth factors released from the corresponding non-functional precursors were also found [12].

In the moss *P.patens* secretome, peptides from membrane and secreted proteins, lipoproteins, pectinesterase-related proteins and cucumsin—a subtilisin-like serine protease—were identified [15,16]. According to the GO enrichment analysis of the moss secreted precursors, most of the proteins were involved in the modification of the cell wall (pectin degradation), extracellular or extrinsic membrane proteins. In addition, proteins participating in photosynthetic reactions, including some chloroplast-coding proteins, such as photosystem I and photosystem II proteins and RUBISCO subunits, were identified [15,16].

**Figure 4:**
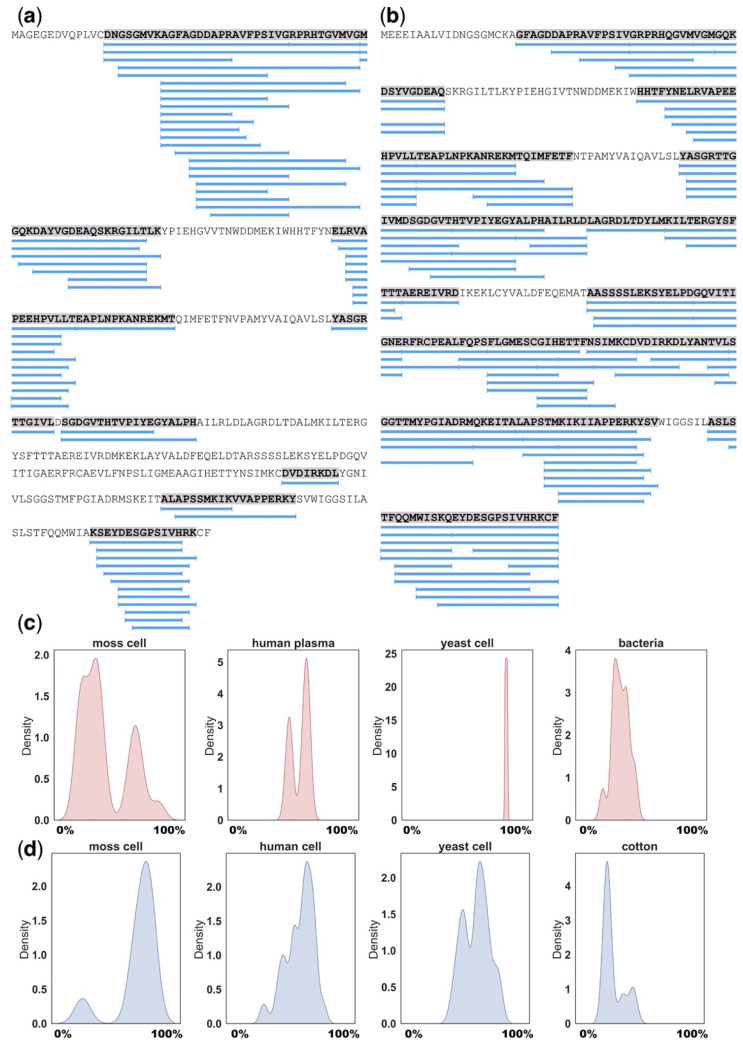
Examples of functional protein degradation patterns. (**a**) MS-identified naturally occurring peptides from *P.patens* actin (UniProt ID: A9TBG2) and (**b**) human actin (UniProt ID: P63261); density plot showing the MS-based degradation patterns of (**c**) mitochondrial ATP synthase subunit and (**d**) glyceraldehyde-3-phosphate dehydrogenase proteins from the moss *P.patens*, human, yeast *Saccharomyces cerevisiae*, bacteria *Lactococcus lactis* and cotton *Gossypium arboreum*. These patterns are presented as distribution of occurrence frequency of peptides per corresponding precursor. The positions of each identified peptide were normalized to protein lengths and represented as percentages. The steps of visualizing and analyzing the data in all figures are available in the GitHub code repository: https://github.com/IgorFesenko/Peptidome_review.

Using BLAST similarity search (E-value < 0.00001; identity > 60%), we found orthologs of precursors from different peptidomic datasets (Table 1). According to our results, the most common protein precursors that had orthologs in plant, human, yeast and bacteria datasets were ATP synthase subunit from mitochondria, glyceraldehyde-3-phosphate dehydrogenase, elongation factor 1-alpha, enolase, heat shock protein, actin, adenosylhomocysteinase, 60S ribosomal protein, S-adenosylmethionine synthetase, fructose-bisphosphate aldolase, histone H2B. Several of the identified homologous precursors have given rise to similar degradation patterns in phylogenetically distant species, as in the case with actin from moss *P. patens* (Figure 4a) and human (Figure 4b). On the contrary, contrast patterns were observed for mitochondrial ATP synthase subunit (Figure 4c) and glyceraldehyde-3-phosphate dehydrogenase (Figure 4d) from moss *P. patens*, human, yeast *S. cerevisiae*, bacteria *L. lactis* and cotton *G. arboreum*. Taken together, published data indicate that the generation of peptide pools appears to be a more deliberate process than chaotic degradation and that the conserved proteins tend to produce stable pools of natively occurring peptides from similar regions (Figure 4). It may be speculated that peptides from functional proteins are generated in the two-step degradation process, in which precursors are primarily divided into relatively large fragments, presumably by proteasomes, followed by further proteolysis into smaller structurally related peptides [93].

A substantial portion of peptides in peptidomes of different organisms originated from proteins with unknown functions. However, it is currently unknown if such peptides are prone to be a source material for further selection and evolution into bioactive peptides (“Raw material” in Figure 1).

## 5. Biological Function of Different Peptide Groups

According to the mechanism of their generation, bioactive peptides can be divided into several groups: peptide hormones and stress-regulating peptides that are released from functional or non-functional protein precursors by specific proteases; those that are derived from functional proteins through proteasomal degradation or by non-specific proteases; and peptides/microproteins, translated directly from small open reading frames (Figure 1). Each group of peptides demonstrates specific activity, for example, through binding to a specific receptor or interacting with functional proteins or small molecules.

Peptide hormones are released from specific, mostly non-functional, precursors and play regulatory roles in all living organisms, from bacteria or fungi to plants and animals [19,69,123,124,125,126]. They are often secreted in extracellular space to perform their functions. For example, five secreted peptides that participate in quorum sensing were found in the bacterium Bacillus subtilis [127,128]. One of them, CSF peptide, is released from the C-terminus of protein precursor PhrC by extracellular serine peptidase and re-enters into the cell to fulfill its function [127,129]. In animals, known peptide hormones are divided into two large groups: growth factors and endocrine hormones, such as neuropeptides [71,130,131,132]. The most well-known examples of animal peptide hormones include insulin, endorphin, gastrin, cholecystokinin (CCK) [71,133,134,135,136], epidermal growth factor (EGF), transforming growth factor beta (TGF-beta), insulin-like growth factor (IGF) and others [130,137,138,139,140,141]. Additionally, there is a group of tissue-specific peptide hormones in animals, such as vasopressin, oxytocin, and bradykinin, that are derived from precursors that additionally contain functional protein sequences [73,74,142,143,144]. The cleavage of precursors occurs under special conditions to yield these peptides [145].

Although the number of known plant bioactive peptides is significantly less than in animals, they have been shown to be important regulators of numerous cellular processes [19,69,106,146,147,148,149,150]. Plant peptide hormones regulate growth and development along with known non-peptide hormones [108]. The most common peptide involved in immune and stress signaling that was found in different plant species is plant elicitor peptide (PEP) [151]. It was shown that PEPs are cleaved from their precursors by metacaspases under an influx of Ca2+ in the cytosol as a rapid response to wounding or pathogen attack [86]. 

Peptide hormones act as ligands for cognate receptors in various organisms, thereby activating cascades of downstream reactions, including protein phosphorylation, and induce the expression of corresponding genes [136,152]. For example, in bacteria, virulence factor production is regulated through the detection of cyclic autoinducing peptides (AIP) by cell-surfaced histidine kinase AgrC [153,154,155]. Overall, the pheromone-receptor systems in Gram-positive bacteria are divided into the following groups: the RNPP (Rap, NprR, PlcR, and PrgX) family of regulators; agr-type cyclic peptides recognized by a two-component signal transduction system (TCSTS), consisting of a histidine kinase, AgrC, and a cytoplasmic response regulator AgrA; the Gly-Gly-type peptide family also recognized by TCSTS, for example, competence-stimulating peptides (CSPs) and their receptors ComD; and the Rgg regulators family, binding sex pheromones, such as sigX-inducing peptide (XIP) and its receptor ComR [125]. 

Overall, receptor kinases for members of 10 plant peptide families have been identified to date [156]. Well-known examples of plant peptide–receptor pairs that mediate growth and development processes are CLAVATA 3 (CLV3)/EMBRYO SURROUNDING REGION (CLE) and their cognate receptor, CLAVATA1 [157]; C-TERMINALLY ENCODED PEPTIDE (CEP) and CEP RECEPTOR [158]; cysteine-rich peptides RAPID ALKALINIZATION FACTOR (RALFs), whose binding to the Catharanthus roseus RLK1-like (CrRLK1L) receptor family and a number of co-receptors, such as FERONIA (FER), ANXUR (ANX)1, ANX2, BUDDHA’S PAPER SEAL (BUPS)1 and BUPS2, and proteins of LORELEI (LRE)-LIKE GLYCOSYLPHOSPHATIDYLINOSITOL (GPI)-ANCHORED PROTEIN (LLG1, LLG2 and LLG3) family mediates cell growth as well as immune responses [107,159,160,161,162].

In yeast, a mating peptide pheromone “a-factor”, the binding of which to a specific receptor Ste3 induces mating processes, was reported to be cleaved out from its precursor by a conserved zinc metalloprotease Ste24, the homologs of which have been found in mammals [126].

In animals, the membrane proteins, referred to as G-protein-coupled receptors (GPCRs), make up the superfamily of receptors responsible for binding with the corresponding peptide ligands and transducing the signal into the cell [163,164,165]. The known network of peptide ligands and GPCRs spans 407 interactions between 219 peptides and 138 receptors in human [166]. For example, growth factors are recognized by specialized receptor tyrosine kinases, such as epidermal growth factor receptors (EGFRs) [167] and platelet-derived growth factor receptor alpha (PDGFRα) [168]. The endothelin signaling peptides bind to their respective endothelin receptors ETA, ETB1, ETB2 and ETC [163]. 

Another group of biologically active peptides—cryptides—that are derived from functional proteins have been found in different organisms [39]. In plants, there are several examples, such as immune peptide GmSubPep (Glycine max Subtilase Peptide), derived from subtilisin-like protease, or an immune peptide CAPE1 (CAP-derived peptide 1) from PR1 protein, and a defense peptide inceptin, cleaved from a plant ATP synthase in larvae of *Spodoptera frugiperda* [40,41,42]. These peptides participate in immune responses. Examples of mammalian cryptides are also known, such as mitocryptide-1 cleaved out from cytochrome c oxidase, which acts as an activator of neutrophils [47], or a peptide hidden in the sequence of proteinase activated receptor 1 (PAR1) named parstatin, with an antagonizing activity to its precursor [169]. A number of cryptides have been discovered, which were cleaved out from hemoglobin precursors not only in blood or the heart, but also in brain tissue [43,44,45,46]. Another example of a known cryptide is a short peptide named EL28, hidden in the 19S ATPase regulatory subunit 4 sequence, increased in abundance upon interferon treatment in human cells. This peptide influences the activities of proteasomes in vitro and was reported to increase the effect of interferon in cells [48]. A peptide derived from histone H2B type 1-H, a PepH, was found in the human brain tissue of schizophrenia patients. It was shown that it participates in protection from cell death [170]. Another example is the peptide Pep5 derived from cyclin D2, which influences cell death in different types of tumor cells [171,172].

Depending on the location and the type of transcripts, smORFs can be classified as short CDSs, intergenic-smORFs, lncRNA-smORFs, or upstream and downstream smORFs [117]. Most of the intergenic smORFs are probably not translated and non-functional [173]. Nevertheless, smORFs have been shown to be a source of functional peptides, regulating key processes in cells [117,174]. Well-studied examples of functional peptides or microproteins encoded by short CDSs are some classes of antimicrobial peptides (AMP), which have been found in a range of organisms from bacteria to plants and animals [175]. Such peptides possess specific physicochemical properties, such as a positive net charge, promoting disruption of cell membrane [176]. In mammals, cysteine-rich β-defensins and histidine-rich histatins are the most studied examples of such peptides [177,178]. Plants also have homologs of mammalian defensins that are encoded by short CDS [179]. Peptides encoded by lncRNAs are the least studied component of peptide pools, but this group may potentially include thousands of peptides [174]. The functional analysis of peptides encoded by lncRNA transcripts was mainly performed on animals [117]. For example, a 46 aa myoregulin (MLN) interacts with sarcoplasmic reticulum Ca2+-ATPase (SERCA) protein in the membrane of the sarcoplasmic reticulum and regulates Ca2+ handling in muscles [180]. Another example is a 53 aa conserved peptide HOXB-AS3, encoded by lncRNA HOXB-AS3, that suppresses colon cancer (CRC) growth [181]. In comparison with animals, the functions of peptides encoded by lncRNAs in plants are not well-studied. There are examples of plant smORF-encoded peptides characterized to date. These are 36-aa POLARIS (PLS) [182], 53-aa ROTUNDIFOLIA4 (ROT4) [183], 51-aa ROT18/DLV1 [184], EARLY NODULIN GENE 40 (ENOD40; 12-, 24-aa) [185], 25-aa KISS OF DEATH (KOD) [186] and 10-aa OSIP108 [187]. Recently, four lncRNA-encoded peptides were characterized in the model plant *Physcomitrium (Physcomitrella) patens* [27]. The overexpression or knockout of these peptides affects plant growth, suggesting their growth-regulating functions [27]. Thus, smORF-encoded peptides may constitute a significant part of cellular and secreted peptidomes, and further studies are needed to understand the abundance, properties, lifetime and functions of such peptides. 

Peptidomes may be a source of molecules for a rapid response to stress or pathogen attack. For example, novel peptides with potential antimicrobial activity derived from functional proteins were found in moss cells and secretomes treated with stress hormones [15,60]. Recent data also indicate that organellar proteases are responsible for the regulation of the generation of stress-signaling peptides [61]. The knockout of oligopeptidases PreP1/2 and OOP triggered the accumulation of peptides, activating a defense response in *Arabidopsis thaliana* [61]. A similar effect has been demonstrated in mice by knockout of thimet oligopeptidase (THOP1), which is reported to be a downstream participant of MHC-bound antigen peptides generation after proteasomal cleavage [104].

## 6. Conclusions

Recent progress in mass-spectrometry-based analysis has expanded our knowledge/view of the composition of intra- and extracellular peptidomes. Besides thousands of newly identified peptides, peptidomic data indicate that intracellular and extracellular degradation of functional proteins is not random and bioactive peptides may be embedded in their sequences. Analysis of the degradation patterns of conserved proteins from different organisms allows us to speculate on the inevitable nature of this process. However, mass-spectrometry analysis has some disadvantages for full peptidome characterization, such as (1) problems with the detection of low-abundance peptides; (2) bias towards the detection of only peptides with certain physicochemical properties; (3) incomplete genome annotations, which require further improvements; and (4) difficulty in correctly identifying modified native peptides. Therefore, further progress is needed to improve the detection of naturally occurring peptides and exclude artifacts during sample preparation. Even more important is the development of approaches for the identification and functional analysis of previously uncharacterized components of cellular and secreted peptidomes.

## Figures and Tables

**Table 1:** Peptidome datasets selected for analysis.

Organism	Number of Peptides	Source	Reference
Human	5548	Blood plasma	[12]
Bacteria *Lactococcus lactis*	1800	Extracellular space	[63]
Cotton *Gossypium arboretum*	1321	Root cells	[62]
Human	4144	Proteasome-associated intracellular peptides	[64]
Yeast *Saccharomyces cerevisiae*	297	Cells	[65]
Moss *Physcomitrium* (*Physcomitrella) patens*	4533 482	Cells Extracellular space	[15]
Moss *Physcomitrium (Physcomitrella) patens*	624	Extracellular space	[16]

## Data Availability

The data analyzed in this study are openly available, references are cited in Table 1.

## References

[1] Wilson I., Vogel J., Somerville S. (1997). Signalling pathways: A common theme in plants and animals?. Curr. Biol..

[2] Matsubayashi Y. (2018). Exploring peptide hormones in plants: Identification of four peptide hormone-receptor pairs and two post-translational modification enzymes. Proc. Jpn. Acad. Ser. B Phys. Biol. Sci..

[3] Pires-daSilva A., Sommer R.J. (2003). The evolution of signalling pathways in animal development. Nat. Rev. Genet..

[4] Rho J.-Y., Yu K., Han J.-S., Chae J.-I., Koo D.-B., Yoon H.-S., Moon S.-Y., Lee K.-K., Han Y.-M. (2006). Transcriptional profiling of the developmentally important signalling pathways in human embryonic stem cells. Hum. Reprod. Oxf. Engl..

[5] Xiao H., Shao F., Wu M., Ren W., Xiong X., Tan B., Yin Y. (2015). The application of antimicrobial peptides as growth and health promoters for swine. J. Anim. Sci. Biotechnol..

[6] Li Y.L., Dai X.R., Yue X., Gao X.-Q., Zhang X.S. (2014). Identification of small secreted peptides (SSPs) in maize and expression analysis of partial SSP genes in reproductive tissues. Planta.

[7] Ivanov V.T., Yatskin O.N., Sazonova O.V., Tolmazova A.G., Leontiev K.V., Philippova M.M., Karelin A.A., Blishchenko E.Y. (2006). Peptidomics: A modern approach to biodiversity of peptides. Pure Appl. Chem..

[8] Tammen H., Schulte I., Hess R., Menzel C., Kellmann M., Mohring T., Schulz-Knappe P. (2005). Peptidomic analysis of human blood specimens: Comparison between plasma specimens and serum by differential peptide display. Proteomics.

[9] Zheng X., Baker H., Hancock W.S. (2006). Analysis of the low molecular weight serum peptidome using ultrafiltration and a hybrid ion trap-Fourier transform mass spectrometer. J. Chromatogr. A.

[10] Fricker L.D. (2010). Analysis of mouse brain peptides using mass spectrometry-based peptidomics: Implications for novel functions ranging from non-classical neuropeptides to microproteins. Mol. Biosyst..

[11] Teixeira C.M.M., Correa C.N., Iwai L.K., Ferro E.S., de Castro L.M. (2019). Characterization of Intracellular Peptides from Zebrafish (*Danio rerio*) Brain. Zebrafish.

[12] Parker B.L., Burchfield J.G., Clayton D., Geddes T.A., Payne R.J., Kiens B., Wojtaszewski J.F.P., Richter E.A., James D.E. (2017). Multiplexed Temporal Quantification of the Exercise-regulated Plasma Peptidome *. Mol. Cell. Proteomics.

[13] Ziganshin R., Arapidi G., Azarkin I., Zaryadieva E., Alexeev D., Govorun V., Ivanov V. (2011). New method for peptide desorption from abundant blood proteins for plasma/serum peptidome analyses by mass spectrometry. J. Proteom..

[14] Ziganshin R.H., Ivanova O.M., Lomakin Y.A., Belogurov A.A., Kovalchuk S.I., Azarkin I.V., Arapidi G.P., Anikanov N.A., Shender V.O., Piradov M.A. (2016). The Pathogenesis of the Demyelinating Form of Guillain-Barre Syndrome (GBS): Proteo-peptidomic and Immunological Profiling of Physiological Fluids. Mol. Cell. Proteom..

[15] Fesenko I., Azarkina R., Kirov I., Kniazev A., Filippova A., Grafskaia E., Lazarev V., Zgoda V., Butenko I., Bukato O. (2019). Phytohormone treatment induces generation of cryptic peptides with antimicrobial activity in the Moss Physcomitrella patens. BMC Plant Biol..

[16] Filippova A., Lyapina I., Kirov I., Zgoda V., Belogurov A., Kudriaeva A., Ivanov V., Fesenko I. (2019). Salicylic acid influences the protease activity and posttranslation modifications of the secreted peptides in the moss Physcomitrella patens. J. Pept. Sci..

[17] De Araujo C.B., Heimann A.S., Remer R.A., Russo L.C., Colquhoun A., Forti F.L., Ferro E.S. (2019). Intracellular Peptides in Cell Biology and Pharmacology. Biomolecules.

[18] Klein T., Eckhard U., Dufour A., Solis N., Overall C.M. (2018). Proteolytic Cleavage—Mechanisms, Function, and “Omic” Approaches for a Near-Ubiquitous Posttranslational Modification. Chem. Rev..

[19] Farrokhi N., Whitelegge J.P., Brusslan J.A. (2008). Plant peptides and peptidomics. Plant Biotechnol. J..

[20] Rape M., Jentsch S. (2002). Taking a bite: Proteasomal protein processing. Nat. Cell Biol..

[21] Sahu I., Glickman M.H. (2021). Structural Insights into Substrate Recognition and Processing by the 20S Proteasome. Biomolecules.

[22] Wadler C.S., Vanderpool C.K. (2007). A dual function for a bacterial small RNA: SgrS performs base pairing-dependent regulation and encodes a functional polypeptide. Proc. Natl. Acad. Sci. USA.

[23] Hanada K., Higuchi-Takeuchi M., Okamoto M., Yoshizumi T., Shimizu M., Nakaminami K., Nishi R., Ohashi C., Iida K., Tanaka M. (2013). Small open reading frames associated with morphogenesis are hidden in plant genomes. Proc. Natl. Acad. Sci. USA.

[24] Hashimoto Y., Ito Y., Niikura T., Shao Z., Hata M., Oyama F., Nishimoto I. (2001). Mechanisms of neuroprotection by a novel rescue factor humanin from Swedish mutant amyloid precursor protein. Biochem. Biophys. Res. Commun..

[25] Guo B., Zhai D., Cabezas E., Welsh K., Nouraini S., Satterthwait A.C., Reed J.C. (2003). Humanin peptide suppresses apoptosis by interfering with Bax activation. Nature.

[26] Hashimoto Y., Kondo T., Kageyama Y. (2008). Lilliputians get into the limelight: Novel class of small peptide genes in morphogenesis. Dev. Growth Differ..

[27] Fesenko I., Kirov I., Kniazev A., Khazigaleeva R., Lazarev V., Kharlampieva D., Grafskaia E., Zgoda V., Butenko I., Arapidi G. (2019). Distinct types of short open reading frames are translated in plant cells. Genome Res..

[28] Guerra-Almeida D., Tschoeke D.A., Nunes-da-Fonseca R. (2021). Understanding small ORF diversity through a comprehensive transcription feature classification. DNA Res. Int. J. Rapid Publ. Rep. Genes Genomes.

[29] VanOrsdel C.E., Kelly J.P., Burke B.N., Lein C.D., Oufiero C.E., Sanchez J.F., Wimmers L.E., Hearn D.J., Abuikhdair F.J., Barnhart K.R. (2018). Identifying New Small Proteins in Escherichia coli. Proteomics.

[30] Ma J., Ward C.C., Jungreis I., Slavoff S.A., Schwaid A.G., Neveu J., Budnik B.A., Kellis M., Saghatelian A. (2014). Discovery of Human sORF-Encoded Polypeptides (SEPs) in Cell Lines and Tissue. J. Proteome Res..

[31] Fuchs S., Kucklick M., Lehmann E., Beckmann A., Wilkens M., Kolte B., Mustafayeva A., Ludwig T., Diwo M., Wissing J. (2021). Towards the characterization of the hidden world of small proteins in Staphylococcus aureus, a proteogenomics approach. PLOS Genet..

[32] Schlesinger D., Elsässer S.J. (2021). Revisiting sORFs: Overcoming challenges to identify and characterize functional microproteins. FEBS J..

[33] McManus I.R. (1958). Synthesis of intracellular peptides in *Torula utilis*. J. Biol. Chem..

[34] Connell G.E., Watson R.W. (1957). Intracellular peptides of Pseudomonas hydrophila. Biochim. Biophys. Acta.

[35] Caron E., Kowalewski D.J., Chiek Koh C., Sturm T., Schuster H., Aebersold R. (2015). Analysis of Major Histocompatibility Complex (MHC) Immunopeptidomes Using Mass Spectrometry. Mol. Cell. Proteom. MCP.

[36] Ruiz Cuevas M.V., Hardy M.-P., Hollý J., Bonneil É., Durette C., Courcelles M., Lanoix J., Côté C., Staudt L.M., Lemieux S. (2021). Most non-canonical proteins uniquely populate the proteome or immunopeptidome. Cell Rep..

[37] Brunet M.A., Lucier J.-F., Levesque M., Leblanc S., Jacques J.-F., Al-Saedi H.R.H., Guilloy N., Grenier F., Avino M., Fournier I. (2021). OpenProt 2021: Deeper functional annotation of the coding potential of eukaryotic genomes. Nucleic Acids Res..

[38] Delcourt V., Brunelle M., Roy A.V., Jacques J.-F., Salzet M., Fournier I., Roucou X. (2018). The Protein Coded by a Short Open Reading Frame, Not by the Annotated Coding Sequence, Is the Main Gene Product of the Dual-Coding Gene MIEF1. Mol. Cell. Proteom..

[39] Autelitano D.J., Rajic A., Smith A.I., Berndt M.C., Ilag L.L., Vadas M. (2006). The cryptome: A subset of the proteome, comprising cryptic peptides with distinct bioactivities. Drug Discov. Today.

[40] Pearce G., Munske G., Yamaguchi Y., Ryan C.A. (2010). Structure-activity studies of GmSubPep, a soybean peptide defense signal derived from an extracellular protease. Peptides.

[41] Chen Y.-L., Lee C.-Y., Cheng K.-T., Chang W.-H., Huang R.-N., Nam H.G., Chen Y.-R. (2014). Quantitative Peptidomics Study Reveals That a Wound-Induced Peptide from PR-1 Regulates Immune Signaling in Tomato. Plant Cell.

[42] Schmelz E.A., Carroll M.J., LeClere S., Phipps S.M., Meredith J., Chourey P.S., Alborn H.T., Teal P.E.A. (2006). Fragments of ATP synthase mediate plant perception of insect attack. Proc. Natl. Acad. Sci. USA.

[43] Ivanov V.T., Karelin A.A., Philippova M.M., Nazimov I.V., Pletnev V.Z. (1997). Hemoglobin as a source of endogenous bioactive peptides: The concept of tissue-specific peptide pool. Biopolymers.

[44] Karelin A.A., Filippova M.M., Iatskin O.N., Blishchenko E.I., Nazimov I.V., Ivanov V.T. (1998). Proteolytic degradation of hemoglobin in erythrocytes results in formation of biologically active peptides. Bioorgan. Khim..

[45] Gomes I., Dale C.S., Casten K., Geigner M.A., Gozzo F.C., Ferro E.S., Heimann A.S., Devi L.A. (2010). Hemoglobin-derived Peptides as Novel Type of Bioactive Signaling Molecules. AAPS J..

[46] Gelman J.S., Sironi J., Castro L.M., Ferro E.S., Fricker L.D. (2010). Hemopressins and other hemoglobin-derived peptides in mouse brain: Comparison between brain, blood, and heart peptidome and regulation in Cpefat/fat mice. J. Neurochem..

[47] Mukai H., Hokari Y., Seki T., Takao T., Kubota M., Matsuo Y., Tsukagoshi H., Kato M., Kimura H., Shimonishi Y. (2008). Discovery of Mitocryptide-1, a Neutrophil-activating Cryptide from Healthy Porcine Heart. J. Biol. Chem..

[48] Monte E.R.C., Rossato C., Llanos R.P., Russo L.C., de Castro L.M., Gozzo F.C., de Araujo C.B., Peron J.P.S., Sant’Anna O.A., Ferro E.S. (2017). Interferon-gamma activity is potentiated by an intracellular peptide derived from the human 19S ATPase regulatory subunit 4 of the proteasome. J. Proteom..

[49] Iavarone F., Desiderio C., Vitali A., Messana I., Martelli C., Castagnola M., Cabras T. (2018). Cryptides: Latent peptides everywhere. Crit. Rev. Biochem. Mol. Biol..

[50] Samir P., Link A.J. (2011). Analyzing the Cryptome: Uncovering Secret Sequences. AAPS J..

[51] Wieczorek M., Abualrous E.T., Sticht J., Álvaro-Benito M., Stolzenberg S., Noé F., Freund C. (2017). Major Histocompatibility Complex (MHC) Class I and MHC Class II Proteins: Conformational Plasticity in Antigen Presentation. Front. Immunol..

[52] Tang D., Kang R., Coyne C.B., Zeh H.J., Lotze M.T. (2012). PAMPs and DAMPs: Signal 0 s that spur autophagy and immunity. Immunol. Rev..

[53] Pouwels S.D., Heijink I.H., ten Hacken N.H.T., Vandenabeele P., Krysko D.V., Nawijn M.C., van Oosterhout A.J.M. (2014). DAMPs activating innate and adaptive immune responses in COPD. Mucosal Immunol..

[54] Schaefer L. (2014). Complexity of danger: The diverse nature of damage-associated molecular patterns. J. Biol. Chem..

[55] Yatim N., Cullen S., Albert M.L. (2017). Dying cells actively regulate adaptive immune responses. Nat. Rev. Immunol..

[56] Dinarello C.A. (2018). Overview of the IL-1 family in innate inflammation and acquired immunity. Immunol. Rev..

[57] Boller T., Felix G. (2009). A renaissance of elicitors: Perception of microbe-associated molecular patterns and danger signals by pattern-recognition receptors. Annu. Rev. Plant Biol..

[58] Anthoney N., Foldi I., Hidalgo A. (2018). Toll and Toll-like receptor signalling in development. Development.

[59] Ramada M.H.S., Brand G.D., Abrão F.Y., Oliveira M., Filho J.L.C., Galbieri R., Gramacho K.P., Prates M.V., Bloch C. (2017). Encrypted Antimicrobial Peptides from Plant Proteins. Sci. Rep..

[60] Khazigaleeva R.A., Vinogradova S.V., Petrova V.L., Fesenko I.A., Arapidi G.P., Kamionskaya A.M., Govorun V.M., Ivanov V.T. (2017). Antimicrobial activity of endogenous peptides of the moss Physcomitrella patens. Russ. J. Bioorg. Chem..

[61] Kmiec B., Branca R.M.M., Berkowitz O., Li L., Wang Y., Murcha M.W., Whelan J., Lehtiö J., Glaser E., Teixeira P.F. (2018). Accumulation of endogenous peptides triggers a pathogen stress response in Arabidopsis thaliana. Plant J..

[62] Yuan N., Dai C., Ling X., Zhang B., Du J. (2019). Peptidomics-based study reveals that GAPEP1, a novel small peptide derived from pathogenesis-related (PR) protein of cotton, enhances fungal disease resistance. Mol. Breed..

[63] Guillot A., Boulay M., Chambellon É., Gitton C., Monnet V., Juillard V. (2016). Mass Spectrometry Analysis of the Extracellular Peptidome of Lactococcus lactis: Lines of Evidence for the Coexistence of Extracellular Protein Hydrolysis and Intracellular Peptide Excretion. J. Proteome Res..

[64] Wolf-Levy H., Javitt A., Eisenberg-Lerner A., Kacen A., Ulman A., Sheban D., Dassa B., Fishbain-Yoskovitz V., Carmona-Rivera C., Kramer M.P. (2018). Revealing the cellular degradome by mass spectrometry analysis of proteasome-cleaved peptides. Nat. Biotechnol..

[65] Dasgupta S., Yang C., Castro L.M., Tashima A.K., Ferro E.S., Moir R.D., Willis I.M., Fricker L.D. (2016). Analysis of the Yeast Peptidome and Comparison with the Human Peptidome. PLoS ONE.

[66] Süssmuth R.D., Mainz A. (2017). Nonribosomal Peptide Synthesis—Principles and Prospects. Angew. Chem. Int. Ed..

[67] Schardon K., Hohl M., Graff L., Pfannstiel J., Schulze W., Stintzi A., Schaller A. (2016). Precursor processing for plant peptide hormone maturation by subtilisin-like serine proteinases. Science.

[68] Magalhães B., Trindade F., Barros A.S., Klein J., Amado F., Ferreira R., Vitorino R. (2018). Reviewing Mechanistic Peptidomics in Body Fluids Focusing on Proteases. Proteomics.

[69] Ryan C.A., Pearce G., Scheer J., Moura D.S. (2002). Polypeptide Hormones. Plant Cell.

[70] Harris R.B. (1989). Processing of pro-hormone precursor proteins. Arch. Biochem. Biophys..

[71] Douglass J., Civelli O., Herbert E. (1984). Polyprotein gene expression: Generation of diversity of neuroendocrine peptides. Annu. Rev. Biochem..

[72] Pottathil M., Lazazzera B.A. (2003). The extracellular Phr peptide-Rap phosphatase signaling circuit of *Bacillus subtilis*. Front. Biosci. J. Virtual Libr..

[73] Coates L.C., Birch N.P. (1998). Differential Cleavage of Provasopressin by the Major Molecular Forms of SPC3. J. Neurochem..

[74] Burbach J.P., Lebouille J.L. (1983). Proteolytic conversion of arginine-vasopressin and oxytocin by brain synaptic membranes. Characterization of formed peptides and mechanisms of proteolysis. J. Biol. Chem..

[75] Patel N., Mohd-Radzman N.A., Corcilius L., Crossett B., Connolly A., Cordwell S.J., Ivanovici A., Taylor K., Williams J., Binos S. (2018). Diverse Peptide Hormones Affecting Root Growth Identified in the Medicago truncatula Secreted Peptidome. Mol. Cell. Proteom..

[76] Hook V., Funkelstein L., Lu D., Bark S., Wegrzyn J., Hwang S.-R. (2008). Proteases for Processing Proneuropeptides into Peptide Neurotransmitters and Hormones. Annu. Rev. Pharmacol. Toxicol..

[77] Olsson V., Joos L., Zhu S., Gevaert K., Butenko M.A., De Smet I. (2019). Look Closely, the Beautiful May Be Small: Precursor-Derived Peptides in Plants. Annu. Rev. Plant Biol..

[78] Corbière A., Vaudry H., Chan P., Walet-Balieu M.-L., Lecroq T., Lefebvre A., Pineau C., Vaudry D. (2019). Strategies for the Identification of Bioactive Neuropeptides in Vertebrates. Front. Neurosci..

[79] Le Gall S.M., Meneton P., Mauduit P., Dreux C. (2004). The sequential cleavage of membrane anchored pro-EGF requires a membrane serine protease other than kallikrein in rat kidney. Regul. Pept..

[80] Tsumagari K., Chang C.-H., Ishihama Y. (2021). Exploring the landscape of ectodomain shedding by quantitative protein terminomics. IScience.

[81] Weber S., Saftig P. (2012). Ectodomain shedding and ADAMs in development. Development.

[82] Beard H.A., Barniol-Xicota M., Yang J., Verhelst S.H.L. (2019). Discovery of Cellular Roles of Intramembrane Proteases. ACS Chem. Biol..

[83] Tian M., Win J., Song J., van der Hoorn R., van der Knaap E., Kamoun S. (2007). A Phytophthora infestans Cystatin-Like Protein Targets a Novel Tomato Papain-Like Apoplastic Protease. Plant Physiol..

[84] Fagundes D., Bohn B., Cabreira C., Leipelt F., Dias N., Bodanese-Zanettini M.H., Cagliari A. (2015). Caspases in plants: Metacaspase gene family in plant stress responses. Funct. Integr. Genom..

[85] Liu H., Hu M., Wang Q., Cheng L., Zhang Z. (2018). Role of Papain-Like Cysteine Proteases in Plant Development. Front. Plant Sci..

[86] Hander T., Fernández-Fernández Á.D., Kumpf R.P., Willems P., Schatowitz H., Rombaut D., Staes A., Nolf J., Pottie R., Yao P. (2019). Damage on plants activates Ca2+-dependent metacaspases for release of immunomodulatory peptides. Science.

[87] Funkelstein L., Lu W.D., Koch B., Mosier C., Toneff T., Taupenot L., O’Connor D.T., Reinheckel T., Peters C., Hook V. (2012). Human Cathepsin V Protease Participates in Production of Enkephalin and NPY Neuropeptide Neurotransmitters. J. Biol. Chem..

[88] Tornero P., Conejero V., Vera P. (1996). Primary structure and expression of a pathogen-induced protease (PR-P69) in tomato plants: Similarity of functional domains to subtilisin-like endoproteases. Proc. Natl. Acad. Sci. USA.

[89] Fricker L.D., Gelman J.S., Castro L.M., Gozzo F.C., Ferro E.S. (2012). Peptidomic analysis of HEK293T cells: Effect of the proteasome inhibitor epoxomicin on intracellular peptides. J. Proteome Res..

[90] Gelman J.S., Sironi J., Berezniuk I., Dasgupta S., Castro L.M., Gozzo F.C., Ferro E.S., Fricker L.D. (2013). Alterations of the Intracellular Peptidome in Response to the Proteasome Inhibitor Bortezomib. PLoS ONE.

[91] Dasgupta S., Castro L.M., Dulman R., Yang C., Schmidt M., Ferro E.S., Fricker L.D. (2014). Proteasome Inhibitors Alter Levels of Intracellular Peptides in HEK293T and SH-SY5Y Cells. PLoS ONE.

[92] Kanlaya R., Thongboonkerd V. (2018). Quantitative peptidomics of endogenous peptides involved in TGF-β1-induced epithelial mesenchymal transition of renal epithelial cells. Cell Death Discov..

[93] Fesenko I.A., Arapidi G.P., Skripnikov A.Y., Alexeev D.G., Kostryukova E.S., Manolov A.I., Altukhov I.A., Khazigaleeva R.A., Seredina A.V., Kovalchuk S.I. (2015). Specific pools of endogenous peptides are present in gametophore, protonema, and protoplast cells of the moss *Physcomitrella patens*. BMC Plant Biol..

[94] Lecker S.H., Goldberg A.L., Mitch W.E. (2006). Protein Degradation by the Ubiquitin–Proteasome Pathway in Normal and Disease States. J. Am. Soc. Nephrol..

[95] Reits E., Griekspoor A., Neijssen J., Groothuis T., Jalink K., van Veelen P., Janssen H., Calafat J., Drijfhout J.W., Neefjes J. (2003). Peptide Diffusion, Protection, and Degradation in Nuclear and Cytoplasmic Compartments before Antigen Presentation by MHC Class I. Immunity.

[96] Ferro E.S., Hyslop S., Camargo A.C.M. (2004). Intracellullar peptides as putative natural regulators of protein interactions. J. Neurochem..

[97] Camargo A.C., Graeff F.G. (1969). Subcellular distribution and properties of the bradykinin inactivation system in rabbit brain homogenates. Biochem. Pharmacol..

[98] Oliveira E.B., Martins A.R., Camargo A.C.M. (1976). Isolation of brain endopeptidases: Influence of size and sequence of substrates structurally related to bradykinin. Biochemistry.

[99] Checler F., Ferro E.S. (2018). Neurolysin: From Initial Detection to Latest Advances. Neurochem. Res..

[100] Camargo A.C.M., Oliveira E.B., Toffoletto O., Metters K.M., Rossier J. (1987). Brain Endo-Oligopeptidase A, a Putative Enkephalin Converting Enzyme. J. Neurochem..

[101] Villar-Cheda B., Dominguez-Meijide A., Valenzuela R., Granado N., Moratalla R., Labandeira-Garcia J.L. (2014). Aging-related dysregulation of dopamine and angiotensin receptor interaction. Neurobiol. Aging.

[102] Berti D.A., Morano C., Russo L.C., Castro L.M., Cunha F.M., Zhang X., Sironi J., Klitzke C.F., Ferro E.S., Fricker L.D. (2009). Analysis of Intracellular Substrates and Products of Thimet Oligopeptidase in Human Embryonic Kidney 293 Cells. J. Biol. Chem..

[103] Russo S.J., Murrough J.W., Han M.-H., Charney D.S., Nestler E.J. (2012). Neurobiology of resilience. Nat. Neurosci..

[104] Santos N.B.D., Franco R.D., Camarini R., Munhoz C.D., Eichler R.A.S., Gewehr M.C.F., Reckziegel P., Llanos R.P., Dale C.S., da Silva V.R.O. (2019). Thimet Oligopeptidase (EC 3.4.24.15) Key Functions Suggested by Knockout Mice Phenotype Characterization. Biomolecules.

[105] Teixeira P.F., Kmiec B., Branca R.M.M., Murcha M.W., Byzia A., Ivanova A., Whelan J., Drag M., Lehtiö J., Glaser E. (2017). A multi-step peptidolytic cascade for amino acid recovery in chloroplasts. Nat. Chem. Biol..

[106] Chen Y.-L., Fan K.-T., Hung S.-C., Chen Y.-R. (2020). The role of peptides cleaved from protein precursors in eliciting plant stress reactions. New Phytol..

[107] Tavormina P., De Coninck B., Nikonorova N., De Smet I., Cammue B.P.A. (2015). The Plant Peptidome: An Expanding Repertoire of Structural Features and Biological Functions. Plant Cell.

[108] Stührwohldt N., Schaller A. (2019). Regulation of plant peptide hormones and growth factors by post-translational modification. Plant Biol..

[109] Rehfeld J.F., Bardram L., Cantor P., Cerman J., Hilsted L., Johnsen A.H., Mogensen N., Osdum L. (1989). Peptide Hormone Expression and Precursor Processing. Acta Oncol..

[110] Harno E., Gali Ramamoorthy T., Coll A.P., White A. (2018). POMC: The Physiological Power of Hormone Processing. Physiol. Rev..

[111] Peng J., Zhang H., Niu H., Wu R. (2020). Peptidomic analyses: The progress in enrichment and identification of endogenous peptides. TrAC Trends Anal. Chem..

[112] Ferro E.S., Rioli V., Castro L.M., Fricker L.D. (2014). Intracellular peptides: From discovery to function. EuPA Open Proteom..

[113] Charneski C.A., Hurst L.D. (2014). Positive Charge Loading at Protein Termini Is Due to Membrane Protein Topology, Not a Translational Ramp. Mol. Biol. Evol..

[114] Kyte J., Doolittle R.F. (1982). A simple method for displaying the hydropathic character of a protein. J. Mol. Biol..

[115] Piovesana S., Capriotti A.L., Cerrato A., Crescenzi C., La Barbera G., Laganà A., Montone C.M., Cavaliere C. (2019). Graphitized Carbon Black Enrichment and UHPLC-MS/MS Allow to Meet the Challenge of Small Chain Peptidomics in Urine. Anal. Chem..

[116] Fesenko I., Shabalina S.A., Mamaeva A., Knyazev A., Glushkevich A., Lyapina I., Ziganshin R., Kovalchuk S., Kharlampieva D., Lazarev V. (2021). A vast pool of lineage-specific microproteins encoded by long non-coding RNAs in plants. Nucleic Acids Res..

[117] Couso J.-P., Patraquim P. (2017). Classification and function of small open reading frames. Nat. Rev. Mol. Cell Biol..

[118] Vakirlis N., Acar O., Hsu B., Castilho Coelho N., Van Oss S.B., Wacholder A., Medetgul-Ernar K., Bowman R.W., Hines C.P., Iannotta J. (2020). De novo emergence of adaptive membrane proteins from thymine-rich genomic sequences. Nat. Commun..

[119] Chen Z., Zhao P., Li F., Leier A., Marquez-Lago T.T., Wang Y., Webb G.I., Smith A.I., Daly R.J., Chou K.-C. (2018). iFeature: A Python package and web server for features extraction and selection from protein and peptide sequences. Bioinform. Oxf. Engl..

[120] Cock P.J.A., Antao T., Chang J.T., Chapman B.A., Cox C.J., Dalke A., Friedberg I., Hamelryck T., Kauff F., Wilczynski B. (2009). Biopython: Freely available Python tools for computational molecular biology and bioinformatics. Bioinformatics.

[121] Mamaeva A., Taliansky M., Filippova A., Love A.J., Golub N., Fesenko I. (2020). The role of chloroplast protein remodeling in stress responses and shaping of the plant peptidome. New Phytol..

[122] Lyapina I., Filippova A., Kovalchuk S., Ziganshin R., Mamaeva A., Lazarev V., Latsis I., Mikhalchik E., Panasenko O., Ivanov O. (2021). Possible role of small secreted peptides (SSPs) in immune signaling in bryophytes. Plant Mol. Biol..

[123] Nässel D.R., Zandawala M. (2019). Recent advances in neuropeptide signaling in *Drosophila*, from genes to physiology and behavior. Prog. Neurobiol..

[124] Monnet V., Juillard V., Gardan R. (2016). Peptide conversations in Gram-positive bacteria. Crit. Rev. Microbiol..

[125] Cook L.C., Federle M.J. (2014). Peptide pheromone signaling in Streptococcus and Enterococcus. FEMS Microbiol. Rev..

[126] Michaelis S., Barrowman J. (2012). Biogenesis of the Saccharomyces cerevisiae Pheromone a-Factor, from Yeast Mating to Human Disease. Microbiol. Mol. Biol. Rev..

[127] Lazazzera B.A. (2001). The intracellular function of extracellular signaling peptides. Peptides.

[128] Pottathil M., Jung A., Lazazzera B.A. (2008). CSF, a Species-Specific Extracellular Signaling Peptide for Communication among Strains of Bacillus subtilis and Bacillus mojavensis. J. Bacteriol..

[129] Lanigan-Gerdes S., Briceno G., Dooley A.N., Faull K.F., Lazazzera B.A. (2008). Identification of Residues Important for Cleavage of the Extracellular Signaling Peptide CSF of Bacillus subtilis from Its Precursor Protein. J. Bacteriol..

[130] Massagué J., Pandiella A. (1993). Membrane-anchored growth factors. Annu. Rev. Biochem..

[131] Elphick M.R., Mirabeau O., Larhammar D. (2018). Evolution of neuropeptide signalling systems. J. Exp. Biol..

[132] Takei Y., Ando H., Tsutsui K. (2016). Handbook of Hormones.

[133] Vigna S.R. (2000). Evolution of the Cholecystokinin and Gastrin Peptides and Receptors1. Am. Zool..

[134] Petersen M.C., Shulman G.I. (2018). Mechanisms of Insulin Action and Insulin Resistance. Physiol. Rev..

[135] Mao X.-F., Wu H.-Y., Tang X.-Q., Ali U., Liu H., Wang Y.-X. (2019). Activation of GPR40 produces mechanical antiallodynia via the spinal glial interleukin-10/β-endorphin pathway. J. Neuroinflamm..

[136] Zeng Q., Ou L., Wang W., Guo D.-Y. (2020). Gastrin, Cholecystokinin, Signaling, and Biological Activities in Cellular Processes. Front. Endocrinol..

[137] Dignass A.U., Sturm A. (2001). Peptide growth factors in the intestine. Eur. J. Gastroenterol. Hepatol..

[138] Wrigley S., Arafa D., Tropea D. (2017). Insulin-Like Growth Factor 1: At the Crossroads of Brain Development and Aging. Front. Cell. Neurosci..

[139] Ipsa E., Cruzat V.F., Kagize J.N., Yovich J.L., Keane K.N. (2019). Growth Hormone and Insulin-Like Growth Factor Action in Reproductive Tissues. Front. Endocrinol..

[140] Hawkes C.P., Levitt Katz L.E., Polin R.A., Abman S.H., Rowitch D.H., Benitz W.E. (2017). 143—Growth Factor Regulation of Fetal Growth. Fetal and Neonatal Physiology.

[141] Fabregat I., Caballero-Díaz D. (2018). Transforming Growth Factor-β-Induced Cell Plasticity in Liver Fibrosis and Hepatocarcinogenesis. Front. Oncol..

[142] Acher R., Chauvet J. (1988). Structure, processing and evolution of the neurohypophysial hormone-neurophysin precursors. Biochimie.

[143] Marcos-Contreras O.A., Martinez de Lizarrondo S., Bardou I., Orset C., Pruvost M., Anfray A., Frigout Y., Hommet Y., Lebouvier L., Montaner J. (2016). Hyperfibrinolysis increases blood–brain barrier permeability by a plasmin- and bradykinin-dependent mechanism. Blood.

[144] Yin Y.-L., Ye C., Zhou F., Wang J., Yang D., Yin W., Wang M.-W., Xu H.E., Jiang Y. (2021). Molecular basis for kinin selectivity and activation of the human bradykinin receptors. Nat. Struct. Mol. Biol..

[145] Sharma J.N., Narayanan P., Sharma J.N. (2014). The Kallikrein–Kinin Pathways in Hypertension and Diabetes. Recent Developments in the Regulation of Kinins.

[146] Butenko M.A., Vie A.K., Brembu T., Aalen R.B., Bones A.M. (2009). Plant peptides in signalling: Looking for new partners. Trends Plant Sci..

[147] Hu Z., Zhang H., Shi K. (2018). Plant peptides in plant defense responses. Plant Signal. Behav..

[148] Gancheva M.S., Malovichko Y.V., Poliushkevich L.O., Dodueva I.E., Lutova L.A. (2019). Plant Peptide Hormones. Russ. J. Plant Physiol..

[149] Hsiao Y.-C., Yamada M. (2021). The Roles of Peptide Hormones and Their Receptors during Plant Root Development. Genes.

[150] Hirakawa Y., Sawa S. (2019). Diverse function of plant peptide hormones in local signaling and development. Curr. Opin. Plant Biol..

[151] Huffaker A., Pearce G., Ryan C.A. (2006). An endogenous peptide signal in Arabidopsis activates components of the innate immune response. Proc. Natl. Acad. Sci. USA.

[152] Czyzewicz N., Yue K., Beeckman T., De Smet I. (2013). Message in a bottle: Small signalling peptide outputs during growth and development. J. Exp. Bot..

[153] Novick R.P., Geisinger E. (2008). Quorum Sensing in *Staphylococci*. Annu. Rev. Genet..

[154] Dunman P.M., Murphy E., Haney S., Palacios D., Tucker-Kellogg G., Wu S., Brown E.L., Zagursky R.J., Shlaes D., Projan S.J. (2001). Transcription Profiling-Based Identification of Staphylococcus aureus Genes Regulated by the agr and/or sarA Loci. J. Bacteriol..

[155] Koenig R.L., Ray J.L., Maleki S.J., Smeltzer M.S., Hurlburt B.K. (2004). *Staphylococcus aureus* AgrA Binding to the RNAIII-agr Regulatory Region. J. Bacteriol..

[156] Furumizu C., Krabberød A.K., Hammerstad M., Alling R.M., Wildhagen M., Sawa S., Aalen R.B. (2021). The sequenced genomes of nonflowering land plants reveal the innovative evolutionary history of peptide signaling. Plant Cell.

[157] Hazak O., Hardtke C.S. (2016). CLAVATA 1-type receptors in plant development. J. Exp. Bot..

[158] Chapman K., Ivanovici A., Taleski M., Sturrock C.J., Ng J.L.P., Mohd-Radzman N.A., Frugier F., Bennett M.J., Mathesius U., Djordjevic M.A. (2020). CEP receptor signalling controls root system architecture in Arabidopsis and Medicago. New Phytol..

[159] Xiao Y., Stegmann M., Han Z., DeFalco T.A., Parys K., Xu L., Belkhadir Y., Zipfel C., Chai J. (2019). Mechanisms of RALF peptide perception by a heterotypic receptor complex. Nature.

[160] Stegmann M., Monaghan J., Smakowska-Luzan E., Rovenich H., Lehner A., Holton N., Belkhadir Y., Zipfel C. (2017). The receptor kinase FER is a RALF-regulated scaffold controlling plant immune signaling. Science.

[161] Du C., Li X., Chen J., Chen W., Li B., Li C., Wang L., Li J., Zhao X., Lin J. (2016). Receptor kinase complex transmits RALF peptide signal to inhibit root growth in *Arabidopsis*. Proc. Natl. Acad. Sci. USA.

[162] Krause C., Richter S., Knöll C., Jürgens G. (2013). Plant secretome—From cellular process to biological activity. Biochim. Biophys. Acta.

[163] Marín-García J., Marín-García J. (2014). Chapter 3—Post-Genomics Cardiovascular Signaling Pathways. Post-Genomic Cardiology.

[164] Krumm B.E., Grisshammer R. (2015). Peptide ligand recognition by G protein-coupled receptors. Front. Pharmacol..

[165] Davenport A.P., Scully C.C.G., de Graaf C., Brown A.J.H., Maguire J.J. (2020). Advances in therapeutic peptides targeting G protein-coupled receptors. Nat. Rev. Drug Discov..

[166] Foster S.R., Hauser A.S., Vedel L., Strachan R.T., Huang X.-P., Gavin A.C., Shah S.D., Nayak A.P., Haugaard-Kedström L.M., Penn R.B. (2019). Discovery of Human Signaling Systems: Pairing Peptides to G Protein-Coupled Receptors. Cell.

[167] An Z., Aksoy O., Zheng T., Fan Q.-W., Weiss W.A. (2018). Epidermal growth factor receptor and EGFRvIII in glioblastoma: Signaling pathways and targeted therapies. Oncogene.

[168] Hunziker M., O’Donnell A.-M., Puri P. (2017). Platelet-derived growth factor receptor alpha-positive cells: A new cell type in the human ureteropelvic junction. Pediatr. Res..

[169] Zania P., Gourni D., Aplin A.C., Nicosia R.F., Flordellis C.S., Maragoudakis M.E., Tsopanoglou N.E. (2009). Parstatin, the cleaved peptide on proteinase-activated receptor 1 activation, is a potent inhibitor of angiogenesis. J. Pharmacol. Exp. Ther..

[170] Café-Mendes C.C., Ferro E.S., Torrão A.S., Crunfli F., Rioli V., Schmitt A., Falkai P., Britto L.R., Turck C.W., Martins-de-Souza D. (2017). Peptidomic analysis of the anterior temporal lobe and corpus callosum from schizophrenia patients. J. Proteom..

[171] De Araujo C.B., de Lima L.P., Calderano S.G., Silva Damasceno F., Silber A.M., Elias M.C. (2019). Pep5, a Fragment of Cyclin D2, Shows Antiparasitic Effects in Different Stages of the Trypanosoma cruzi Life Cycle and Blocks Parasite Infectivity. Antimicrob. Agents Chemother..

[172] De Araujo C.B., Russo L.C., Castro L.M., Forti F.L., do Monte E.R., Rioli V., Gozzo F.C., Colquhoun A., Ferro E.S. (2014). A novel intracellular peptide derived from g1/s cyclin d2 induces cell death. J. Biol. Chem..

[173] Andrews S.J., Rothnagel J.A. (2014). Emerging evidence for functional peptides encoded by short open reading frames. Nat. Rev. Genet..

[174] Chen Y., Li D., Fan W., Zheng X., Zhou Y., Ye H., Liang X., Du W., Zhou Y., Wang K. (2020). PsORF: A database of small ORFs in plants. Plant Biotechnol. J..

[175] Buda De Cesare G., Cristy S.A., Garsin D.A., Lorenz M.C. (2020). Antimicrobial Peptides: A New Frontier in Antifungal Therapy. MBio.

[176] Rios A.C., Moutinho C.G., Pinto F.C., Del Fiol F.S., Jozala A., Chaud M.V., Vila M.M.D.C., Teixeira J.A., Balcão V.M. (2016). Alternatives to overcoming bacterial resistances: State-of-the-art. Microbiol. Res..

[177] Meade K.G., O’Farrelly C. (2019). β-Defensins: Farming the Microbiome for Homeostasis and Health. Front. Immunol..

[178] Khurshid Z., Najeeb S., Mali M., Moin S.F., Raza S.Q., Zohaib S., Sefat F., Zafar M.S. (2017). Histatin peptides: Pharmacological functions and their applications in dentistry. Saudi Pharm. J. SPJ.

[179] Sher Khan R., Iqbal A., Malak R., Shehryar K., Attia S., Ahmed T., Ali Khan M., Arif M., Mii M. (2019). Plant defensins: Types, mechanism of action and prospects of genetic engineering for enhanced disease resistance in plants. 3 Biotech.

[180] Anderson D.M., Anderson K.M., Chang C.-L., Makarewich C.A., Nelson B.R., McAnally J.R., Kasaragod P., Shelton J.M., Liou J., Bassel-Duby R. (2015). A Micropeptide Encoded by a Putative Long Non-coding RNA Regulates Muscle Performance. Cell.

[181] Huang J.-Z., Chen M., Chen D., Gao X.-C., Zhu S., Huang H., Hu M., Zhu H., Yan G.-R. (2017). A Peptide Encoded by a Putative lncRNA HOXB-AS3 Suppresses Colon Cancer Growth. Mol. Cell.

[182] Casson S.A., Chilley P.M., Topping J.F., Evans I.M., Souter M.A., Lindsey K. (2002). The POLARIS gene of Arabidopsis encodes a predicted peptide required for correct root growth and leaf vascular patterning. Plant Cell.

[183] Narita N.N., Moore S., Horiguchi G., Kubo M., Demura T., Fukuda H., Goodrich J., Tsukaya H. (2004). Overexpression of a novel small peptide ROTUNDIFOLIA4 decreases cell proliferation and alters leaf shape in *Arabidopsis thaliana*. Plant J. Cell Mol. Biol..

[184] Guo P., Yoshimura A., Ishikawa N., Yamaguchi T., Guo Y., Tsukaya H. (2015). Comparative analysis of the RTFL peptide family on the control of plant organogenesis. J. Plant Res..

[185] Röhrig H., Schmidt J., Miklashevichs E., Schell J., John M. (2002). Soybean ENOD40 encodes two peptides that bind to sucrose synthase. Proc. Natl. Acad. Sci. USA.

[186] Blanvillain R., Young B., Cai Y., Hecht V., Varoquaux F., Delorme V., Lancelin J.-M., Delseny M., Gallois P. (2011). The Arabidopsis peptide kiss of death is an inducer of programmed cell death. EMBO J..

[187] De Coninck B., Carron D., Tavormina P., Willem L., Craik D.J., Vos C., Thevissen K., Mathys J., Cammue B.P.A. (2013). Mining the genome of Arabidopsis thaliana as a basis for the identification of novel bioactive peptides involved in oxidative stress tolerance. J. Exp. Bot..

